# Eye and neural defects associated with loss of GDF6

**DOI:** 10.1186/1471-213X-6-43

**Published:** 2006-09-29

**Authors:** Meredith L Hanel, Carmel Hensey

**Affiliations:** 1 UCD School of Biomolecular and Biomedical Science, Conway Institute, University College Dublin, Belfield, Dublin 4, Ireland

## Abstract

**Background:**

In *Xenopus *the bone morphogenetic protein growth and differentiation factor 6 (GDF6) is expressed at the edge of the neural plate, and within the anterior neural plate including the eye fields. Here we address the role of GDF6 in neural and eye development by morpholino knockdown experiments.

**Results:**

We show that depletion of GDF6 (BMP13) resulted in a reduction in eye size, loss of laminar structure and a reduction in differentiated neural cell types within the retina. This correlated with a reduction in staining for Smad1/5/8 phosphorylation indicating a decrease in GDF6 signalling through loss of phosphorylation of these intracellular mediators of bone morphogenetic protein (BMP) signalling. In addition, the Pax6 expression domain is reduced in size at early optic vesicle stages. Neural cell adhesion molecule (NCAM) is generally reduced in intensity along the neural tube, while in the retina and brain discreet patches of NCAM expression are also lost. GDF6 knock down resulted in an increase in cell death along the neural tube and within the retina as determined by terminal deoxynucleotidyl transferase-mediated dUTP nick end labeling (TUNEL) staining.

**Conclusion:**

Our data demonstrate that GDF6 has an important role in neural differentiation in the eye as well as within the central nervous system, and that GDF6 may act in some way to maintain cell survival within the ectoderm, during the normal waves of programmed cell death.

## Background

That BMP signalling controls many essential processes in eye development is evidenced by the fact that disruptions in BMP signalling in many model organisms result in morphologically small or misshapen eyes which have underlying defects in histology and/or neurogenesis [1-5]. BMP's are expressed in morphogenetic gradients throughout the developing embryo. BMP signalling is modulated by BMP antagonists which heterodimerize with BMP's and inhibit binding to their receptors. Initially neural induction occurs in an environment where BMP signalling is blocked by BMP antagonists that are secreted from the Spemann organizer [6,7]. However BMP's are later expressed in neural tissue and are found to promote CNS development [8-11]. Tissues respond differently to different levels of BMP signalling. Dpp, the *Drosophila *homolog of BMP4, operates at different thresholds within the neuroectoderm to either inhibit neurogenesis or promote dorsal fates [12]. Within neural tissues, BMP's have been found to have roles in a wide range of processes such as dorsal-ventral patterning, regulating cell division, apoptosis and setting up axon guidance cues [13].

Gradients of BMP's and BMP antagonists establish dorsal-ventral characteristics within both the neural tube and retina, which develop as bilateral evaginations from the neural tube. In the neural tube many BMP's are expressed dorsally at the roof plate and control region-specific expression of transcription factors that are involved in specification of dorsal and ventral types of neurons [14]. In *Xenopus *overexpression of BMP4 in the retina causes expansion of dorsal retina markers, while overexpression of its antagonist noggin causes expansion of ventral retina markers [15]. Antagonistic relationships between BMP4, BMP2 and the antagonist ventroptin in chick retina establish proper expression of axon guidance molecules [16,17].

BMP signalling in the eye is important for the establishment of domains of Pax6 and Pax2 which demarcate the optic cup and optic stalk respectively [15]. Pax6 is one of the earliest markers expressed in the eye field [18] and plays a key role in maintaining multipotency of neuronal cells [19]. BMP4 expressed dorsally and sonic hedgehog (*Shh*) expressed ventrally have opposing effects on proximal-distal and dorsal-ventral properties of the developing retina impacting on Pax2 and Pax6 [15,20].

Growth and differentiation factors (GDF's) are a subgroup within the bone morphogenetic proteins. GDF's are able to heterodimerize with BMP's and signal through the same Smads as BMP's [21,22]. Phylogenetic analysis places GDF6 (*BMP13*) into a subgroup, that also contains GDF5 and GDF7 (BMP12) [22], which are involved in development of joints and cartilage [23-26]. GDF7 was shown to promote differentiation of a discrete class of dorsal interneurons in mouse [8]. The zebrafish homolog to GDF6, *Radar*, was shown to have a role in maintenance of neuroectodermal identity [27,28].

GDF6 expression in *Xenopus *as well as the homolog *radar *in zebrafish have been described [21,27]. In *Xenopus *neurulation GDF6 is expressed at the edges of the neural plate, within the anterior neural plate and eye fields. After neural tube closure GDF6 is expressed in the neural tube and retina expression becomes restricted to the dorsal side. The conserved expression pattern of GDF6 in *Xenopus *and zebrafish eye development suggests a conserved developmental function in this tissue. We wished to further investigate the role of GDF6 in neural development and specifically within the retina in *Xenopus*. At late blastula – early gastrula stages of development, *Xenopus *GDF6 has a very restricted expression compared with BMPs 2, 4 and 7. GDF6 expression initially is completely restricted to the animal cap ectoderm, and remains ectodermal throughout development [21], whereas BMP2, BMP4 and BMP7 all have wider expression patterns which include mesoderm [29,30]. Thus we have a unique opportunity with GDF6 to address the role of BMP signalling specifically in ectoderm/neural tissue.

Here we demonstrate that loss of function experiments by morpholino antisense knockdown resulted in reduced expression of a phosphorylated form of Smad in the developing eye. Our GDF6 knock down resulted in neurulation defects in the eye as well as the neural tube, with an underlying loss of Pax6 and NCAM expression. Loss of GDF6 also resulted in increased cell death pointing to a role for GDF6 in retinogenesis that is attributable to a promotion of cell survival during neural differentiation.

## Results

### Reduced eye size following GDF6 depletion

**Figure 1:**
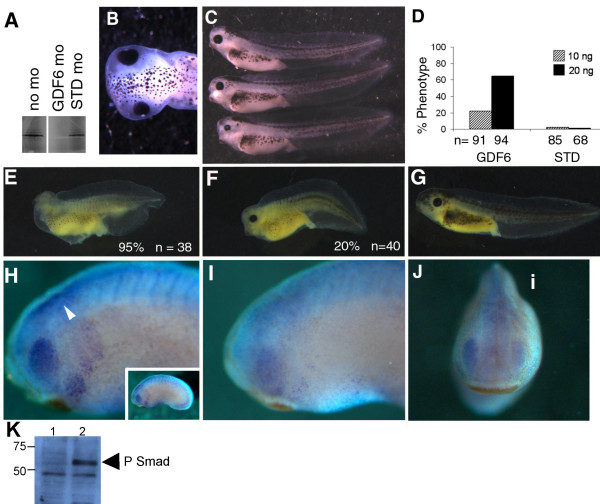
**Reduced eye size following GDF6 depletion**. (A) *In vitro *translation of GDF6 mRNA is blocked by GDF6 MOrpholino (GDF6 MO) but is not affected by the standard control MOrpholino (STD MO). (B) Dorsal view of stage 41 tadpole injected with 10 ng GDF6 MO on the left side. (C) Lateral view of tadpoles showing (top to bottom) an unaffected tadpole; GDF6 MO injected sides with an eye 84% of normal contra lateral size and 66% of normal contra lateral size. (D) Graphical representation of the percent of embryos injected with GDF6 MO with the small eye phenotype at stage 41 compared with those injected with STD MO. (E) A severely ventralized embryo injected with 250 pg GDF6 mRNA at stage 41. The percent of ventralized embryos is shown. (F) An example of a partially rescued embryo injected with 250 pg GDF6 mRNA + 20 ng GDF6 MO. The percent of similarly ventralized embryos is shown. (G) A completely rescued embryo injected with 250 pg GDF6 mRNA + 20 ng GDF6 MO. (H) The untreated side of a stage 24 embryo immunostained with anti-phosphorylated-Smad1/5/8 showing staining within the retina and along the neural tube (arrow). Full embryo is shown in the inset. (I) The GDF6 MO (20 ng) injected side of the same embryo showing a loss of phosphorylated-Smad1/5/8 in the retina and neural tube. (J) Frontal view of the same stage 24 embryo showing the reduction in the intensity of the phosphorylated-Smad1/5/8 stain on the injected side (i). (K) Endogenous Smad1/5/8 phosphorylation in St.11.5–12.5 embryonic extracts, *lane 2*. No Smad phosphorylation is detected in 64 cell embryos *lane 1*.

To test whether GDF6 has a role in eye development, we designed a morpholino against the ATG site of *Xenopus *GDF6. Embryo halves depleted of GDF6 showed a striking reduction in eye size compared to the uninjected side when assessed at stage 41, a time when the major events in eye development are relatively complete (Fig. 1B,1C, and 1D). On average, eye size decreased by 16%–34%. MO treatment had a dose dependent effect on eye size with 22% of embryos displaying the phenotype at the lower 10 ng dose compared to 65% of embryos displaying small eyes at the 20 ng dose (Fig. 1D). A standard MO (STD MO) had no significant effect on eye size even at the highest MO dose injected (Fig. 1D).

In addition to the injection of the STD MO as a control for toxicity, we took a number of approaches to verify the specificity of our morpholino. Firstly, *in vitro*, the GDF6 MO specifically and efficiently blocked translation of its target mRNA, while the STD MO had no effect (Fig. 1A). One way to demonstrate *in vivo *specificity is to rescue the MO induced phenotype by coinjecting mRNA for the target gene. It was not possible to produce a convincing rescue of the small eye phenotype by co-injecting GDF6 mRNA nor the zebrafish homolog *Radar *mRNA along with the GDF6 MO. The results from these experiments were difficult to interpret since both GDF6 mRNA and *Radar *mRNA overexpression caused an overall ventralized phenotype in which the eyes could be small due to either ventralization or the GDF6 MO knock down. This type of rescue experiment is further complicated by the fact that the coinjected mRNA would have effects much earlier and ubiquitously compared with the endogenous GDF6. As an alternative we took advantage of the ventralization effect of the injected GDF6 mRNA, and rescued this by coinjection of the GDF6 MO (Fig. 1E,1F, and 1G). GDF6 mRNA over expression ventralizes embryos, a phenotype that was efficiently rescued by co-injecting GDF6 MO, highlighting the specificity of this MO's activity (Fig. 1E,1F and 1G). 95% (n = 38) of embryos injected with 250 pg GDF6 mRNA were ventralized with DAI values of 1–3 (Fig. 1E). Co-injection of 250 pg GDF6 mRNA with 20 ng GDF6 MO resulted in only 20% (n = 40) of embryos being ventralized and all had less severe DAI values of 3–4 (Fig. 1F). The remaining 80% of embryos had a normal axis (Fig. 1G).

Our third approach for verifying the specificity of our GDF6 MO knock down was to detect a loss in BMP signalling *in vivo*. Studies have shown that treatment of cells with recombinant GDF6 activated the Smad1,5,8 pathway as evidenced by phospho-Smad1/5/8 detection [22]. Analysis of phosho-Smad staining in *Xenopus *by whole mount immunohistochemistry has shown a particularly strong expression in the retina and neural tube from neurulation onwards [37]. Analysis of phospho-Smad1/5/8 in embryo halves injected with GDF6 MO showed a marked reduction in phospho-Smad staining (Fig. 1I), compared to the uninjected half (Fig. 1H). In embryos injected with 20 ng and 50 ng GDF6 MO there was a loss of stain intensity in the retina for phospho-Smad1/5/8 on the injected side in 30% (n = 20) and 57% (n = 21) of embryos respectively. Physically the eye appeared normal in that the lateral bulge was visible even though the stain was reduced in intensity. The reduced intensity of phospho-Smad1/5/8 was more evident on the dorsal side of the retina, fitting with the dorsal-high expression of GDF6 by *in situ *hybridization [21]. In addition we noted a reduction in stain intensity within the neural tube on the injected side, also a site of GDF6 expression [21] (compare Fig. 1I and 1H arrowhead). No loss of phospho-Smad1/5/8 staining was observed in the retina when 50 ng standard morpholino was injected (n = 22) (not shown). The phospho-Smad antibody used specifically detects the expected 60 kDa band of endogenous phospho-Smad in gastrula stage embryonic extracts (Fig. 1K, lane 2). This band was not detected in earlier embryonic extracts where BMP signalling is not yet active (Fig. 1K, lane 1).

We have provided evidence that our GDF6 MO is able to block both *in vitro *translation and *in vivo *translation of GDF6 mRNA. That our GDF6 MO also blocks endogenous GDF6 mRNA *in vivo *is strongly supported by the reduction in signalling through phospho-Smad1/5/8. Together these results show that the small eye phenotype induced by injection of GDF6 MO is caused by depletion of GDF6 and GDF6 mediated BMP signalling.

### Loss of retinal differentiation and laminar structure in GDF6 depleted embryos

**Figure 2:**
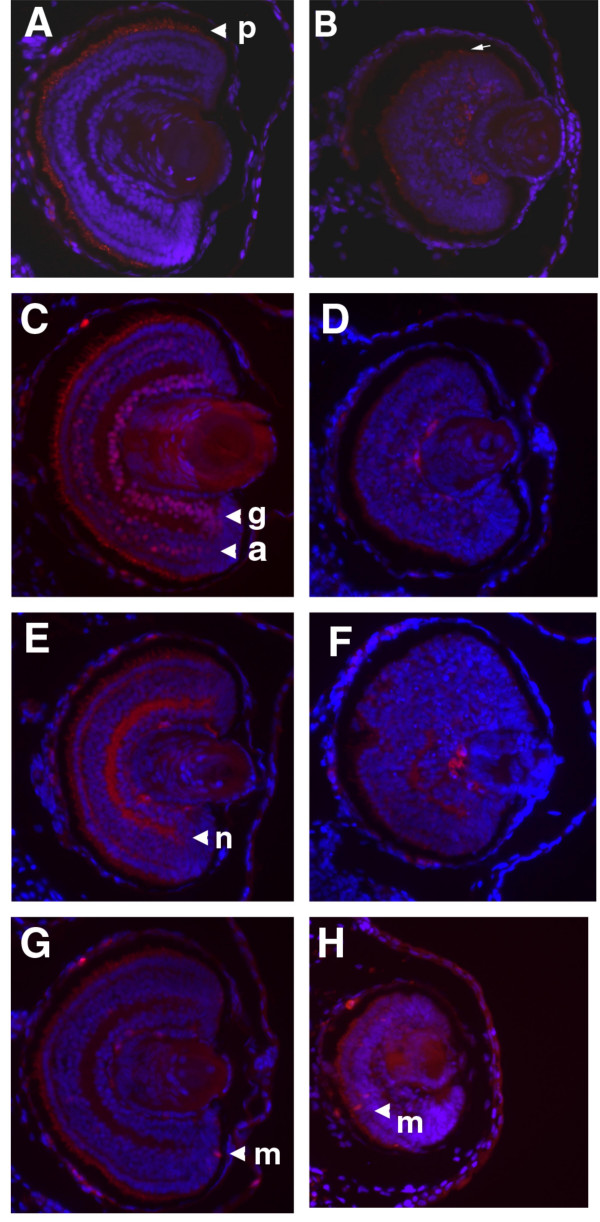
**Loss of retinal differentiation and laminar structure in GDF6 depleted embryos**. Histology of stage 41 embryos with small eyes following GDF6 MO injection (20 ng) compared with normal stage matched controls. DAPI staining of small eye (B, D, F, and H) compared with normal eye (A, C, E, and G) showing small eyes have a lack of laminar structure and appear disorganized. (A, B) Photoreceptors (p) are not stained in B compared with XAP-1 staining in A. (B) Cell bodies are visibly extending outward (white arrow) but do not stain for XAP-1. Red staining next to the lens is non-specific staining also detected in a proportion of negative controls. (C, D) 40.2D6 detects ganglion (g) and amacrine (a) cells within the normal retina, but these cells are not detected in the small eye. Photoreceptors are visible due to autofluorescence at increased exposure. (E, F) Small disorganized eyes show a loss of immunostaining for the cytoplasmic domain of NCAM (n). (G, H) Mitotic cells (m) detected using anti-phosphorylated-histoneH3 are few and found mainly in the ciliary marginal zone in normal stage 41 embryos. The small eye shows a few mitotic cells near the RPE layer.

Histological examination of the small eye phenotype by DAPI staining revealed that the organization of nuclear layers in normal retina (Fig. 2A,2C,2E and 2G) was completely lost in the small eyes (Fig. 2B,2D,2F and 2H). We investigated whether the retinal neurons normally present in the differentiated retina were present in the disorganized retinas of the GDF6 knock down tadpoles by staining for photoreceptors and ganglion and amacrine cells. We found that the small eyes were negative for the photoreceptor marker XAP-1 (compare Fig. 2A and 2B). We did note that cell bodies in the outer layer of the neural retina appeared to extend outward like photoreceptors, even though they did not stain for XAP-1. These extensions might represent an attempt to form photoreceptor cells, however they are not stained for XAP1 which only detects properly assembled outer segment membranes [38]. The anti-islet-1 antibody which detects ganglion and amacrine cells did not detect any of these cells within the small eye (compare Fig. 2C and 2D).

To determine the degree of neural differentiation within the small retinas, we stained for the NCAM cytoplasmic domain which stains the nerve fibres within the retina. The small eyes showed only small patches of NCAM staining (Fig. 2E and 2F), demonstrating that they contained very few mature neurons. At stage 41 the *Xenopus *retina is differentiated, except for the cillary marginal zone (CMZ) regions where undifferentiated cells are still mitotically active. Using phospho-histone H3 as a mitotic marker, we found normal control retinas to contain only one or two positively stained cells per section, which always occurred within the CMZ (Fig. 2G). In the small eyes we found some mitotic cells outside the RPE, but there was no sign of massive increased proliferation within the small eyes. This demonstrated that while the cells in the small retinas did not appear to be mature neurons, in general most of the cells within the retina of the small eyes were post mitotic, but had not differentiated into specific retinal cell types.

### GDF6 knock down disrupts Pax6

**Figure 3:**
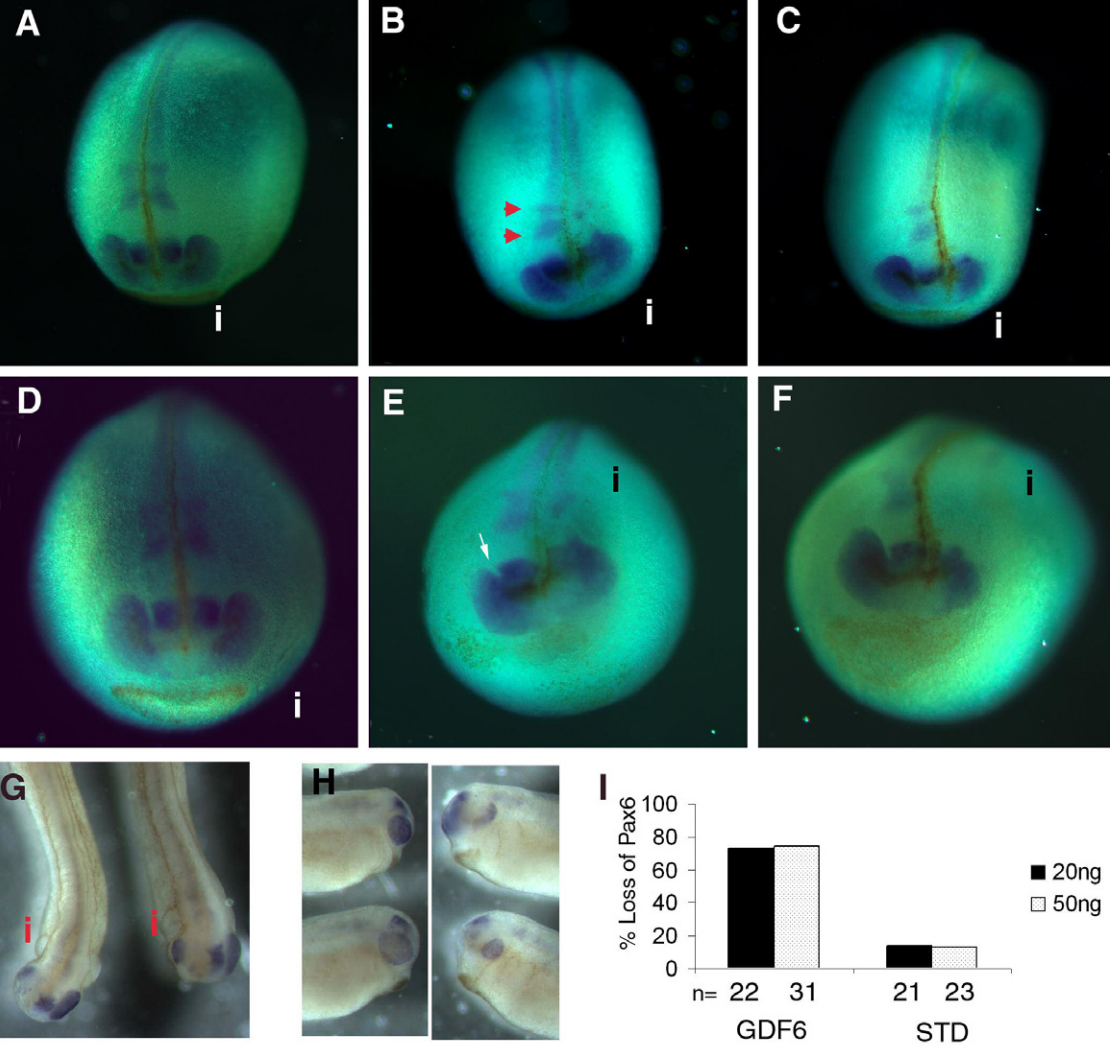
**GDF6 knock down causes a loss of Pax6 expression as detected by *in situ *hybridization**. Representative stage 20 embryos injected with 50 ng standard MO (A, D), 20 ng GDF6 MO (B, E), and 50 ng GDF6 MO (C, F). The injected sides are labelled i. Dorsal views show a loss of Pax6 stain within the two rhombomere bands in the hindbrain in the 20 ng GDF6 MO injected embryos (B), and an additional more severe loss of dorsal neural tube staining in the 50 ng GDF6 MO injected embryos (C), compared to the uninjected side (red arrows indicate rhombomeres) and standard MO injected embryos (A). Frontal views show a laterally reduced size of the Pax6 domain (E and F, injected side), compared to Pax6 staining in uninjected side (definition between forebrain and retinal stain indicated with white arrow) and standard MO injected embryo (D). (G, H) Dorsal and lateral views of stage 27 embryos injected with 25 ng of GDF6 MO show a reduced size of the Pax6 domain within the retina and a complete loss of stain within brain on the injected side. (I) Graph showing the percent stage 20 embryos injected at 20 ng and 50 ng of GDF6 MO and Standard MO showing a disruption of normal Pax6 stain.

Pax6 is a key regulator of eye development which is expressed early in eye development when the brain first evaginates to form optic vesicles [39-41]. Since *Xenopus *GDF6 is also expressed in the eye fields at stage 20 [21], we tested whether loss of GDF6 expression disrupted Pax6. In stage 20 embryos knock down of GDF6 caused a reduction in the size and altered the shape of the Pax6 domain in the retina and forebrain on the GDF6 MO injected side compared with the uninjected side or control MO injected side (Fig. 3A and 3D control MO injected; Fig 3B,3C,3E, and 3F GDF6 MO injected; 3I). The distinction between the forebrain and retina domain is lost and the retina stain does not extend as far laterally (Fig. 3B,3C,3E,3F injected side-i). Pax6 staining within the brain was also disrupted with a loss of the two rhombomeric bands of stain within the hindbrain (Fig. 3B and 3C injected side). The number of embryos with disrupted Pax6 expression within the stage 20 eye field following injection of GDF6 MO was similar at 20 ng and 50 ng but the 50 ng injections produced a more severe effect, with increasingly reduced Pax6 domains in the retina/forebrain as well as along the neural tube (Fig. 3C and 3F). In addition, at 50 ng the injected side of the embryo appears to show an overall reduction in size. GDF6 depleted embryos continued to show defects in Pax6 staining later in development (St 27) with a reduced area or change in shape of Pax6 staining within the retina and a marked loss of Pax6 in the forebrain (Fig. 3G injected side; Fig 3H). Interestingly, while Pax6 staining was disrupted in GDF6 depleted embryos at both stage 20 and stage 27 only specific areas of Pax6 staining were affected indicating discreet regions of Pax6 are responsive to/dependent on GDF6 signalling. Furthermore, we did not observe a decreased intensity in Pax6, but rather a complete loss of stain in specific areas. Where Pax6 staining remained, such as the more proximal retina stain at stage 20, and parts of the retina at stage 27, staining remained quite intense, indicating an all or nothing response to GDF6 depletion.

### GDF6 knock down disrupts neural differentiation

**Figure 4:**
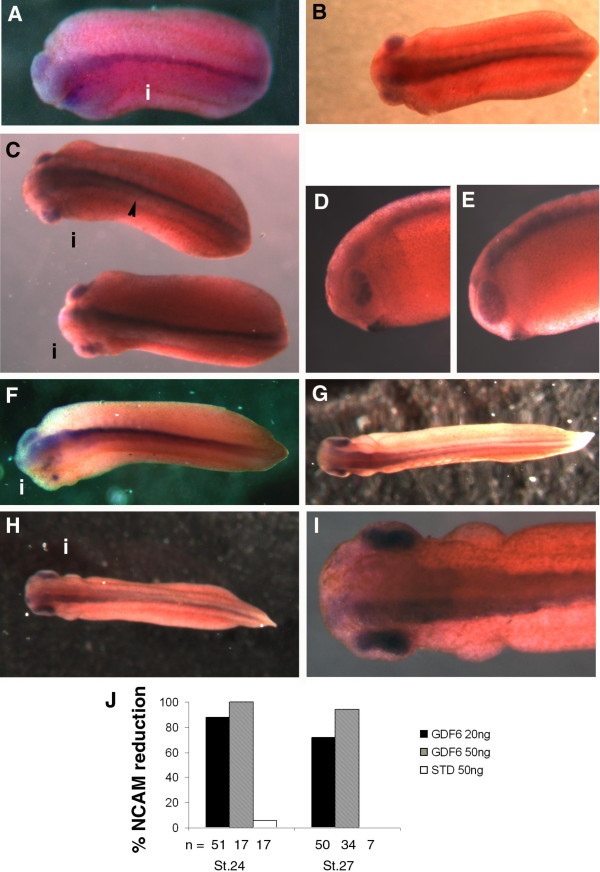
**GDF6 knockdown disrupts neural differentiation**. At neurulation stages, immunostaining for neural cell adhesion molecule (NCAM) is lost or reduced in intensity in the GDF6 MO injected side: A(i), C(i), D, F(i), H(i), I. (A) Stage 24 embryo injected with 50 ng GDF6 MO showing complete loss of NCAM stain along the neural tube on the injected side. Some patchy retina stain remains. This embryo is also curved and appears to be reduced in size on the injected side. (B) Normal NCAM stain in an embryo injected with 50 ng standard MO. (C) Stage 24 embryos injected with 20 ng GDF6 MO with NCAM stain lost along the posterior neural tube (top embryo, arrow marks posterior end of NCAM stain), or along the entire neural tube (bottom embryo). (D, E) Injected (D) and uninjected side (E) of bottom embryo in (C) showing patchy loss of NCAM stain in the retina of the injected side. (F) Dorsal view of stage 27 embryo injected with 50 ng GDF6 MO. The injected side (i) shows a loss of NCAM stain both along the neural tube and within the head and retina. In the head and retina small patches of intense NCAM staining remain. (G) Normal NCAM staining in a stage 27 embryo injected with 50 ng standard MO. (H, I) Full view and close up of a stage 27 embryo injected with 20 ng GDF6 MO. Neural tube NCAM staining is less intense on the injected side (top half of embryo). (J) Percent embryos with NCAM reduction on the injected side following GDF6 MO injection.

Since we detected a loss of mature neurons in the developing retina of GDF6 depleted embryos, using an antibody to NCAM which specifically stains neurons with axons, we extended this analysis of neuronal differentiation from neurula to early tailbud stages. We observed a decrease in intensity of NCAM staining in 72–100% of stage 24–27 embryos injected with 20 ng and 50 ng of GDF6 MO (Fig. 4J). At 50 ng GDF6 MO there was a marked loss of NCAM staining along the neural tube, within the brain and retina (Fig. 4A and 4F compared with 4B and 4G). Within the brain and retina, NCAM staining appeared patchy, whereas the posterior neural tube NCAM staining was reduced uniformly. At the lower dose, 20 ng GDF6 MO, loss of NCAM within the neural tube and the brain was comparatively more severe than loss of NCAM within the retina (Fig. 4C,4H and 4I). A less severe patchy loss of NCAM was detected following 20 ng injections as compared to the 50 ng dose (Fig. 4Ci; 4D injected compared to 4E uninjected). In embryos injected with 20 ng GDF6 MO we found variation in the degree of NCAM reduction in the posterior neural tube. In some cases NCAM loss was confined to the posterior neural tube and in others NCAM was reduced along the entire neural tube (Fig. 4C).

### Increased cell death during neurogenesis in GDF6 depleted embryos

**Figure 5:**
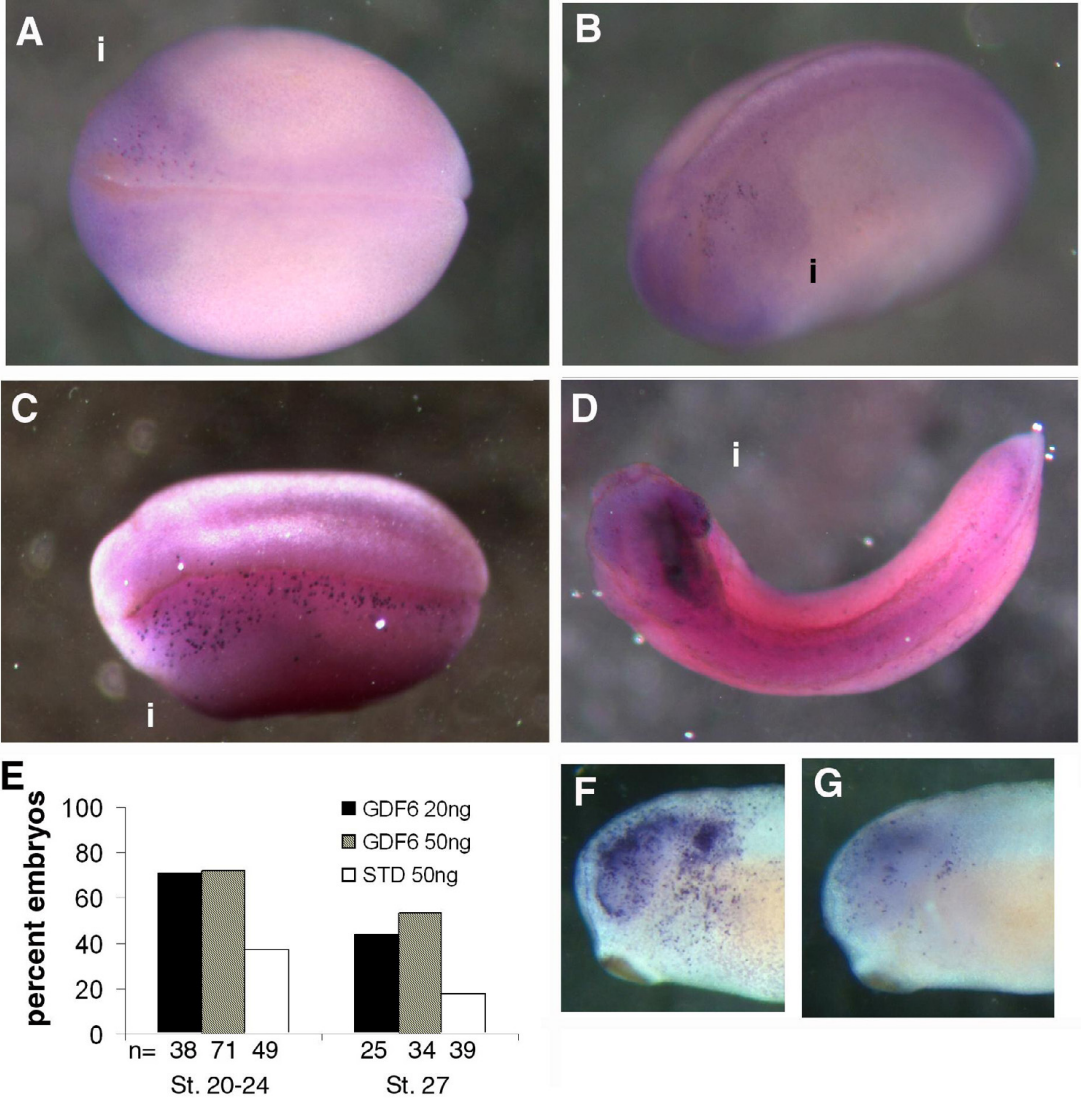
**Increased cell death during neurogenesis in GDF6 depleted embryos**. (A) Stage 20 embryo injected with 20 ng GDF6 MO shows a patch of TUNEL at the anterior region of the injected side (i). (B) Stage 22 embryo injected with 20 ng GDF6 MO showing TUNEL positive cells near the midline at the anterior region of the injected side (i). (C) Stage 22 embryo injected with 50 ng GDF6 MO showing more extensive TUNEL staining which extends along the length of the injected side (i). There is an obvious reduction in physical size on the injected side. (D) Stage 27 embryo injected with 50 ng GDF6 MO showing dark concentrated TUNEL staining in the brain and retina on the injected side (i). This embryo is also curled towards the injected side. (E) Graphical representation of the percent embryos with increased TUNEL staining on the injected side at stage 20–24 and stage 27. (F, G) The GDF6 MO (25 ng) injected side (F) and uninjected side (G) of a stage 27 embryo, showing heavy TUNEL staining in the retina and brain.

The pattern of loss of patches of Pax6 and NCAM stain suggested either a loss of maintenance of expression of these factors or cell death within these tissues. Using TUNEL staining we found that the injected side of stage 20–27 embryos had increased cell death compared to the uninjected side of the same embryo (Fig. 5). When stage 20–24 embryos were analyzed, increased TUNEL staining was found in 34% and 35% more embryos than with standard MO injections in which we observed some background levels of increased TUNEL staining. TUNEL staining mainly occurred adjacent to the midline and in the anterior region, coinciding with areas where PCD naturally occurs during *Xenopus *neurulation [36]. We detected a dose dependent increase in TUNEL positive cells following treatment with GDF6 MO. The number of TUNEL positive cells increased and the area of TUNEL staining was widened and extended posteriorly at the highest MO dose (50 ng) (compare Fig. 5B and 5C). At 20 ng and 50 ng 26% and 35% respectively had increased TUNEL staining above background. The pattern of increased TUNEL at stage 27 was an intense patch within the brain adjacent to the retina and within the retina (Fig. 5D,5F and 5G). Interestingly, this pattern resembles the areas of patchy NCAM staining in stage 24–27 embryos injected with GDF6 MO (Fig. 4D and 4F). We noted that the injected side of the embryos were frequently physically smaller compared with the uninjected side (Fig. 3C,3F, 4A, 5C) or curled towards the injected side (Fig. 5D) which is consistent with a loss of tissue by cell death.

## Discussion

### GDF6 in the context of BMP signalling in eye development

While it had been shown that treatment of cells with recombinant GDF6 lead to phosphorylation of Smad1/5/8 we have shown the first evidence of GDF6 signalling through Smad proteins *in vivo*. GDF6 knockdown lead to a decrease in p-Smad1/5/8 in the eye, neural tube and branchial arches. More specifically, in the developing retina, GDF6 depletion resulted in a specific loss of phospho-Smad1/5/8 in the dorsal retina, an area where GDF6 is expressed, demonstrating that GDF6 has a role in maintaining BMP signalling in the dorsal optic vesicle.

In keeping with the expression of GDF6 within the retina we found that knock down of GDF6 lead to a striking small eye phenotype. Micropthalmia is also associated with BMP4 heterozygote mice, a gene that is also expressed in *Xenopus *developing retina [2,29]. While mouse BMP7 expression is restricted to the RPE and lens, BMP7 knockout mice display a gobal eye developmental defect manifesting as small eyes and anophthalmia [1,4,5]. Additionally these studies now implicate GDF6 (BMP13) in eye development.

Our histological analysis on the GDF6 morpholino induced small eyes showed a marked reduction in NCAM and other retinal neural markers. While we have not conclusively identified the nature of the retinal cells in GDF6 knockdown embryos or whether they are post mitotic or not, the loss of NCAM and other retinal markers coupled with the increased proportion of retinal cells that were undergoing mitosis outside the CMZ, demonstrated that the overall level of terminal differentiation within the small retinas and brain were inhibited. Manipulation of expression of BMP receptors in mouse has shown that BMP signalling has a role in neural differentiation [42,43]. This effect may also be mediated through downstream genes, such as Pax6, which maintains multipotency of retinal progenitor cells [19]. If GDF6 knock down initially reduces the Pax6 field, this could reduce the ability of the retina to differentiate into the various neural cell types. Although both GDF6 and Pax6 expression occupy the entire eye field in a relatively uniform manner at stage 20 we observed only partial loss of Pax6 staining at stage 20 which correlated to the more lateral region. It is possible that other BMP's can compensate for loss of GDF6 in the more medial region of the eye field. Overexpression of BMP4 and the BMP antagonist noggin increase and decrease the domains of Pax6 expression respectively [15].

### GDF6, neural differentiation and cell death

While it is well established that neural induction initially requires blockage of BMP signalling by BMP antagonists [6,7], much work in mamamalian systems has established that later BMP signalling has a positive role in neural development [8-11]. During neurulation GDF6 expression coincides with many regions of active BMP signalling as detected by phospho-Smad1/5/8 particularly in the neural tube and retina [21,37]. The restricted expression of GDF6 to the neuroectoderm allowed us to address the role of BMP signalling in this tissue. We propose that GDF6 has a positive role in neural differentiation within the retina and neural tube and that this may be separate from the epidermal inducing activity that is essential during neural induction. It is likely that heterodimerization between GDF6 and other BMP's such as BMP4 set up temporally and spatially regulated gradients of BMP signalling in neural tissues including the eye.

NCAM is needed for the proper histogenesis of the retina, and since NCAM staining was reduced from early stages of eye development in GDF6 morpholino injected embryos, the abnormal laminar organization that we observed could be directly due to a reduction of NCAM [44]. Fitting with the increased cell death that we found in the NCAM domain, an NCAM-like molecule was found to be protective against apoptosis in neurons [45]. There is some evidence that BMP's can positively regulate cell adhesion molecules [46,47]. The loss of NCAM that we observed more likely demonstrates an inability of neurons to differentiate properly since our NCAM antibody detects NCAM in neurons with axons. In support of this, GDF6, BMP2 and GDF8, each promotes neurite outgrowth when added to retinal ganglion cells in culture [48].

In many organisms including *Xenopus*, developmentally important PCD takes place in actively proliferating neural precursors and new postmitotic neuroblasts [49]. In *Xenopus*, during neurulation PCD has very specific patterns within the brain and sensory placodes and within the neural folds [36]. Much of this PCD appears to occur at the level of neuronal determination [50]. It is becoming clear that some of the same factors that regulate neural development actually regulate PCD [51]. Our data are consistent with GDF6 fitting into this category, as a factor which protects against cell death and also promotes neural differentiation.

A direct link between BMP signalling and apoptosis has not yet been made, however BMP signalling has generally been shown both to promote and inhibit apoptosis in different contexts. In the optic cup of chick, BMP4 and its antagonist noggin have shown pro and anti-apoptotic effects respectively [52]. It has been shown that BMP4 acts to promote survival of newly formed olfactory neurons [11]. In mice Bmpr1b knock out leads to cell death in the retina postnatally at the end of neurogenesis [53]. In conditional double knock outs of Bmpr1a and Bmpr1b in the retina of mice, at retinal neurogenesis stage, there is a marked increase in cell death [42].

Phenotypes described for zebrafish hapoid for a deletion of the *Radar *gene include short axis and reduction in head structures [28]. In addition it was noted that the eyes initially formed but degenerated later, likely due to apoptosis. We similarly observed an increase in apoptosis in the eyes during development however we cannot determine whether the eyes of our GDF6 depleted *Xenopus *embryos grow initially but then later degenerate. In either case, since reduction of GDF6 and haploinsufficiency of zebrafish *Radar *both result in increased cell death in the eye, GDF6/Radar appears to be protective against apoptosis in the retina.

GDF6 may act through Pax6 to regulate cell death. Pax6 mutation is associated with abnormal cell death patterns during development and in differentiation of neurons [54,55]. Decreased expression of Pax6 is associated with apoptotic regression of eyes in the cave dwelling eyeless form of the teleost, *Astyanax mexicanus *compared with its surface dwelling form which has eyes [56]. Further studies have shown that *Shh *and *tiggy-winkle hedgehog (twhh) *gene expression is expanded along the anterior embryonic midline in the cave dwellers and when over-expressed in the surface dwellers, can mimic the eye regression phenotype [57]. Perhaps GDF6 acts in a similar way to BMP4 in opposing Shh to establish proximal-distal and dorsal-ventral properties of the developing retina and in regulating Pax2 and Pax6 domains [15,20].

## Conclusion

We propose that GDF6 is an important early regulator of vertebrate retinal development and likely acts through Pax6 to regulate eye development and subsequently retinal differentiation. This hypothesis is supported by the following points: (1) GDF6 is normally expressed in the developing retina, (2) loss of GDF6 function reduces eye size, (3) loss of GDF6 function disrupts expression of Pax6, (4) loss of GDF6 function leads to reduced neural differentiation as determined by NCAM expression and reduced retinal differentiation as determined by loss of differentiated retinal cell types. Our results suggest that GDF6 may function through regulation of cell death. Loss of GDF6 may lead directly to increased cell death within the neuroectoderm pool of neuronal precursors, resulting in less cells differentiating to neurons. Alternatively, loss of GDF6 leads to a loss of neuronal identity of cells within the neuroectoderm, leading to increased cell death in cells lacking proper determination factors.

## Methods

### Embryological techniques

Embryos were generated by standard techniques as described previously [31,32] and were staged according to Nieukwop and Faber [33]. Microinjections were done in 1 × MMR containing Penicillin-Streptomycin and 3% Ficoll, and injected embryos were cultured in 0.1 × MMR with Penicillin-Streptomycin from pregastrulation to neurulation stage. Embryos were fixed in MEMFA (0.1 M MOPS pH 7.4, 2 mM EDTA, 3.7% Formaldehyde) for 1–2 hours, washed in methanol 2 × 30 minutes and stored at -20°C in methanol.

Embryos were slowly rehydrated in PBSTw 0.2% (0.2% Tween in PBS) for whole mount immunohistochemistry and TUNEL staining (described below) and rehydrated in PBSTw 0.1% for *in situ *hybridization. After staining, samples were refixed with MEMFA overnight at 4°C. Pigmented embryos were bleached either before or after staining as follows. Embryos were incubated in 1% hydrogen peroxide, 5% formamide, 2 × SSC (0.3 M NaCl, 0.03 M sodium citrate, pH 7) for 30 minutes to 1 hour with aluminum foil placed under the vials on a rocker in a well lit area. Stained embryos were processed for photography as follows. Embryos were transferred to methanol 2 × 15 minutes, 10 min PBT (PBS, 0.1% Tween-20, 0.2% BSA), 10 min to 1 hour PBS/Glycerol (1:1) and transferred to glycerol and stored at 4°C. Embryos were analyzed and photographed on a Zeiss Lumar microscope.

### Microinjection of morpholinos and mRNA

The 25 bp GDF6 morpholino (GDF6 MO) consisting of the sequence 5'-gcagagggctcctgtatgtatccat-3' directed at the GDF6 ATG start codon [GenBank:AF155125] and the standard control morpholino (STD MO) were obtained from GeneTools LLC and contained carboxyfluorescein end modifications. Morpholinos were suspended in sterile water. For rescue experiments 250 pg of GDF6 mRNA, 250 pg GDF6 mRNA + 20 ng GDF6 MO and 20 ng GDF6 MO were injected into one cell of two cell embryos. Embryos were sorted into right and left side injected, based on morpholino fluorescence being detected in and restricted to one or other halves of the embryo. Ventralization was assessed using the dorsoanterior index (DAI) [34].

### Synthesis of mRNA and *in vitro *knock down of translation

The GDF6 construct was linearized, and the capped mRNA was synthesized *in vitro *using the mMESSAGE Machine Sp6 kit (Ambion). 1 μg GDF6 mRNA was mixed with 5 μg and 10 μg of either GDF6 MO or 10 μg STD MO. To anneal the morpholino to the target mRNA the mixtures were heated to 70°C for 5 minutes and then allowed to cool gradually over one hour to 37°C. The mRNA and morpholino mixtures were *in vitro *translated in the presence of ^35^S labelled cysteine. The *in vitro *translation products were run on a 10% acrylamide gel, and visualized by X-Ray film.

### Wester blot analysis

Embryos were homogenized (10 μl per embryo) in lysis buffer (20 mM Tris, pH 8, 50 mM NaCl, 10 mM β-glycerophosphate, 2 mM EDTA, 1% NP40, + protease and phosphatase inhibitor cocktail). Lysates were centrifuged for 10 minutes, 4°C, and 6,000 rpm and suspended in 2 × vol Laemmli buffer. They were separated by 10% SDS-PAGE and transferred to nitrocellulose membrane (Protran). For anti phospho-Smad1/5/8 immunoblotting membranes were blocked with 3% BSA in TBST. After several washes in TBST, membranes were incubated overnight with the antibody (1:400). Detection was then done with HRP-labeled secondary antibodies and ECL.

### Whole mount immunohistochemistry

Embryos were washed in PBT (Triton X-100, BSA in PBS) for 15 minutes, followed by 1 hour of blocking in 20% normal goat serum in PBT. Anti-NCAM (4d supernatant-DSHB) was used at 1:20. Anti-Phospo-Smad1/5/8 (Cell Signalling Technologies) was used at 1:500. Antibodies were detected using alkaline phosphatase conjugated secondary antibodies and the chromogenic substrates BCIP (5-Bromo-4-chloro-3-indolyl phosphate, toluidine salt) and NBT (Nitro blue tetrazolium chloride).

### Immunohistochemistry on slides

For immunohistochemistry on slides, sections were dewaxed and hydrated, washed in PBT, and blocked with 20% Normal Goat Serum in PBT. Ganglion and Amacrine cells, Photoreceptor cells, NCAM and mitotic cells were detected with 1:3 anti-islet 1 (40.2D6 supernatant – DSHB), 1:20 anti-XAP (3D2 supernatant – DSHB), 1:20 anti-NCAM (4D supernatant – DSHB) and 1:100 anti-phospho-Histone H3 (Upstate) respectively. Primary antibodies were incubated at room temperature for 1 hour. Slides were washed in PBS 3 × 5 minutes. Secondary Goat anti-mouse-FITC (1:20) and Goat anti-rabbit-rhodamine (1:20) were added and incubated 1 hour at room temperature followed by PBS 2 × 5 min, DAPI in PBS 10 min and 5 min PBS. Slides were mounted with Vectashield (Vector Laboratories Ltd.).

### *In situ *hybridization

*In situ *hybridizations were carried out as previously described [35]. Full length sense and antisense probes were generated using SP6 and T7 *in vitro *transcription (mMESSAGE machine – Ambion) with incorporation of digoxigenin labelled nucleotides. The reaction was fixed with MEMFA overnight at 4°C. Embryos were transferred to methanol 2 × 15 minutes, 10 min PBT, 10 min to 1 hour PBS/Glycerol (1:1) and transferred to glycerol for photographing.

### TUNEL staining

TUNEL staining was done as previously described [36]. Briefly, DNA fragments resulting from apoptosis were end-labelled by incubating embryos in the presence of terminal deoxy transferase (TdT) in the presence of digoxygenin labelled dUTP.

### Embedding and sectioning

Embryos dehydrated in methanol were transferred into Xylene 1 × 5 minutes, 1 × 30 minutes at 60°C. Embryos were transferred to a 1:1 mixture of Xylene: Paraplast 30 min – 1 hr at 60°C. Embryos were transferred to paraplast 3 × 30 minutes at 60°C. Embryos were positioned in moulds, allowed to set, and 10 μm sections were cut. Sections were dewaxed in Xylene substitute (Sigma), hydrated and incubated in DAPI (0.1 mg/ml) in PBS, followed by dehydration in ethanol and mounted with Canada Balsam. For embryos to be used for immunostaining on slides, hydrated sections were stained with antibodies followed by DAPI/PBS and mounted in Vectasheild (Vector Laboratories Ltd.). Slides were analyzed and photographed on a Zeiss Axioplan II microscope.

## Authors' contributions

MH performed the experiments, contributing to experimental design, acquisition, analysis and interpretation of the data. In addition, MH drafted the manuscript. CH contributed to experimental design, interpretation and manuscript preparation. All authors read and approved the final manuscript.
